# Density‐dependent dispersal and habitat use in size‐structured populations: An experiment in wild Trinidadian guppies

**DOI:** 10.1002/ecy.70151

**Published:** 2025-07-18

**Authors:** Sebastiano De Bona, Karendeep Sidhu, Hanna M. Enroth, Andrés López‐Sepulcre

**Affiliations:** ^1^ Department of Biological and Environmental Science University of Jyväskylä Jyväskylä Finland; ^2^ Department of Biology Bryn Mawr College Bryn Mawr Pennsylvania USA; ^3^ School of Biological Sciences University of Aberdeen Aberdeen UK; ^4^ Department of Ecology and Evolutionary Biology Cornell University Ithaca New York USA

**Keywords:** density manipulation experiment, dispersal, dominance, ideal despotic distribution, microhabitat use, *Poecilia reticulata*

## Abstract

Individual responses to density perturbations often depend on individual size or stage, and can include demographic changes (survival, reproduction, growth) and spatial responses (dispersal, habitat shift). While these responses are well characterized, their interaction is seldom considered. Size‐specific effects of density can result from size‐ or stage‐specific spatial responses mediated by dominance interactions: subordinate individuals might suffer disproportionately from overcrowding conditions if displaced from high‐quality habitats by dominant individuals. To investigate this, we performed an experiment in wild guppies where we observed demographic and spatial responses to density manipulations and tested for an interaction between them. We found recruitment, growth, and female survival to be decreased at high density. Dispersal was costly, causing a reduction of body condition and growth. Shifts in microhabitat use with density were size‐dependent: at increased density, large individuals were more likely to remain in a microhabitat, while small individuals were likely to move and suffer reduced growth. At decreased density, growth improved when remaining in the same microhabitat (for large individuals) or moving to a different one (for small individuals). Our results show that regulation under density perturbation can occur through asymmetric interactions that disproportionately affect smaller individuals.

## INTRODUCTION

Density dependence underlies the regulation of populations and can manifest on many aspects of individual life histories. In a system at equilibrium, resources are limiting and population density determines the strength of competition. If density increases, the lower per capita availability of resources is expected to decrease survival (Clutton‐Brock et al., 1985; Einum & Nislow, 2005; Gunnarsson et al., 2006), growth rate (Matthews et al., 2001; Schram et al., 2006; Tanaka et al., 1999; Wilbur, 1977), body condition (McClanahan & Kurtis, 1991; Stewart et al., 2005) or reproduction (Massot et al., 1992), regulating the population back to its original equilibrium (Eisenberg, 1966). In addition, mobile organisms can respond spatially to resource limitation by abandoning a crowded habitat patch for another where per capita resources are more abundant, a phenomenon referred to as positive density‐dependent dispersal (Bowler & Benton, 2005; Lidicker, 1962; Matthysen, 2005).

Spatial responses often entail dispersal movements on a large scale (between habitat patches), but can also emerge at a smaller spatial scale as movement to different microhabitat types (within habitat patch; Bult et al., 1999; Davey et al., 2005). Microhabitat shift might be a quicker, less risky alternative to dispersal (Bonte et al., 2012). If density increases, individuals might be forced into lower quality microhabitat within the same habitat patch, where food sources are less desirable or predation risk is higher but intraspecific competition is weaker. Ideal free distribution theory predicts that if individuals have equal competitive abilities, they will distribute freely in space to equalize the suitability of different habitat patches (Fretwell & Lucas, 1970), and this can be easily extended to lower spatial scales (microhabitat). The suitability of a microhabitat is determined by its inherent properties (food resources, predators, cover) and is modified by how many individuals share that microhabitat. Regardless of the scale at which it is observed, the ideal free distribution hypothesis predicts all individuals will have the same fitness and therefore will be equally affected by density regardless of the (micro)habitat used, because better patches will have higher densities than worse ones (Fretwell & Lucas, 1970).

Despite some good examples in nature (Haché et al., 2013; Haugen et al., 2006), ideal free distributions are unlikely to be commonplace. This can be due to a variety of factors, including the inability to move or to obtain complete information on the available patches, or differences between individuals in their competitive ability. Competitive asymmetries will generate an ideal despotic distribution (Fretwell, 1972), where dominant individuals occupy high‐quality habitats and subordinates are relegated to lower quality ones. As a consequence, when population density increases, it will increase disproportionately in lower quality habitats (Bult et al., 1999; Purchase & Hutchings, 2008), as a result of the subordinate or smaller individuals being displaced. For example, Davey et al. (2005) showed that juvenile bullhead (*Cottus gobio*) were displaced from their preferred habitat by larger adults when density was increased.

Individual traits that cause competitive asymmetries (e.g., size, life stage) are also likely to cause individuals to differ in their responses to density. The Atlantic salmon (*Salmo salar*) can escape the cost of crowding by dispersing away from a high‐density patch when it is at the mobile parr stage, while the less developed fry remain and experience an increase in mortality (Einum et al., 2006). While size and stage‐specific differences in susceptibility to density are well established and replicated in experimental studies (Bassar et al., 2013; Gunnarsson et al., 2006; Massot et al., 1992; Reznick et al., 2012), the potential interaction between spatial responses and life history traits is less explored. If the population follows an ideal despotic distribution, subordinate individuals might be displaced from good quality habitats at high density and pay a cost (in terms of growth and body condition). The effects of density on individual fitness in this case will be mediated by competitive dynamics and size‐specific spatial responses.

When considering spatial responses to density and their fitness consequences, scale should be taken into account (Einum & Nislow, 2005). Density‐dependent dispersal (between habitat patches) and density‐dependent microhabitat use (within a habitat patch) are rarely considered together in the same system (but see Clark et al., 2019), even though they could have distinct consequences on life history. Dispersal is more energetically demanding, potentially reducing survival and growth to a larger degree than microhabitat shift. Dispersal also affects reproduction by changing the pool of potential mates. Density‐dependent microhabitat use, while less costly, might not provide the same magnitude of relief from overcrowding as dispersal would. In order to study size‐specific density effects at multiple spatial scales, one requires a system where (1) density can be easily manipulated, (2) behaviors at small spatial scales are easy to observe, and (3) individual‐based responses can be recorded for different size classes. The Trinidadian guppy (*Poecilia reticulata*) provides such a system. Density regulation and density‐dependent selection are well documented, and adaptations to conspecific density are the proximate cause of the phenotypic differences shown in different predation regimes (Travis et al., 2014, 2023). Observational studies of natural fluctuations in guppy population density show density‐dependent dispersal to differ between newborn and adult guppies (De Bona et al., 2019). Moreover, as population density at the landscape level increases, guppies disperse to suboptimal habitat patches (riffles) at lower local density, where they have similar lifetime reproductive success as in preferred yet crowded pools (Reznick et al., 2020). Despite some evidence for size‐ and sex‐specific microhabitat use (Croft et al., 2003, 2004, 2006; Darden & Croft, 2008), the effects of density at lower spatial scales (microhabitats within a habitat patch) are unknown.

Density manipulation experiments show that guppy populations are density regulated. When population density is artificially perturbed in the wild, guppy populations are quickly regulated back to equilibrium density via alterations of growth, survival, and reproduction (Bassar et al., 2013). The mechanisms of regulation are asymmetric with regard to the direction of density perturbation and differ among size classes (Bassar et al., 2013; Reznick et al., 2012). In a density manipulation study (Reznick et al., 2012) decreased density promoted growth and survival in immature individuals and increased reproductive investment in adult females. Increased density, on the other hand, decreased fat reserves and offspring size in females and increased adult mortality. It is unknown, however, whether these size‐specific responses are evidence of direct competition or differential responses in microhabitat use. This can be understood by observing behaviors at the microhabitat scale during a density perturbation.

In this paper we ask the following questions: (1) How does density perturbation affect dispersal and microhabitat use in guppies? (2) Do these spatial responses have performance consequences? and (3) Do these spatial responses differ among size/stage classes? To answer them, we performed density manipulation experiments in the field. Since natural guppy populations are density regulated (Bassar et al., 2013), we expect increased density to cause survival, growth, and reproduction to decrease (Bassar et al., 2013; Reznick et al., 2012), and the probability to disperse to increase (De Bona et al., 2019). Moreover, if individuals follow an ideal free distribution within a habitat patch, both increasing and decreasing local density might cause individuals to shift to a different microhabitat. If size‐asymmetric effects are absent, all individuals should be more likely to move to a better quality microhabitat when density is decreased, and to a lower quality microhabitat when density is increased. Otherwise, if individuals monopolize resources or hold territories, we should expect size‐specific (or stage‐specific) responses to density perturbations: for example, smaller and younger individuals could be more likely to be displaced to low quality microhabitats at high density, and larger (or more experienced) individuals hold or move to good quality microhabitats at control and low density.

## MATERIALS AND METHODS

### Study species

Guppies (*P. reticulata*) are native to the Caribbean island of Trinidad. They are common in streams of the Northern Range mountains. Guppies are ovoviviparous, giving birth to fry that are 5–6 mm in standard length in our study populations. Brood size varies largely depending on age, usually varying between 2 and 20. In these low‐predation populations, males reach sexual maturity at 57–59 days (14–17 mm) and females at 80–83 days (20–23 mm). Inter‐brood intervals typically range from 25 to 35 days. Guppies are omnivorous and feed on a diet of benthic algae, invertebrates, and detritus (Zandonà et al., 2011).

Several density manipulation experiments have demonstrated that low‐predation populations are tightly density regulated and at their equilibrium densities (λ ≅ 1), and that vital rates respond strongly to density perturbation (Bassar et al., 2013; Reznick et al., 2012).

### Sites and microhabitat types

The study was conducted in spring (March to May) 2018 in the Northern Range mountains of Trinidad. We selected a total of three sites for our experiment: two sites in Caigual and one in Taylor, both tributaries of the Guanapo river. Each site constitutes a section of the stream, between 29 and 33 m long, where several deep, slow‐flowing pools are separated by short, fast‐running riffles. The sections are separated from the rest of the stream by waterfalls both upstream and downstream, which prevents guppies from moving upstream and impedes guppies from climbing into the section from the downstream reaches. The two sites in Caigual were consecutive sections along the stream and shared a barrier waterfall that separated them. In both Caigual and Taylor, the only other species of fish present was *Anablepsoides hartii* (syn. *Rivulus hartii*). Given the lack of major piscivorous predators, all sites were considered to have low‐predation pressure (Endler, 1995). Each site was selected to include at least three similarly sized pools (3–7 m long), hosting a large enough local population of guppies to allow for density manipulation (initial population sizes and densities can be found in Appendix S1) . Pools represented distinct habitat patches (large scale) which included different microhabitat types (small scale). We defined five microhabitat types based on water speed, depth, and benthic substrate. This categorization was done visually by the same observer (S. De Bona) each time for consistency. After classification, we took quantitative habitat measurements (see below) that allowed us to evaluate the consistency of the categories. We defined the five microhabitat categories as follows: the “inflow” of the pool characterized by fast‐running water and coarse substrate; a “beach” represented by a sandy shallow shore with still water; a “core” constituting the central portion of the pool, with relatively slow‐running, deep water, and with depositions of organic matter; a “swamp” representing a marginal part of the pool, with still, shallow water and abundant organic matter; and a “run” consisting of a relatively shallow, fast‐running part of the pool leading to the outflow into the next segment of the stream.

### Capture and marking

At each pool within each site, we visually categorized the microhabitat types present and hand‐sketched a map of their spatial arrangement within the pool. We could keep track of the microhabitat type in which an individual fish was caught by using the sketch as a reference. We fished out each pool using butterfly nets and hand nets until no fish was visible from the surface, transferring all fish captured in the same microhabitat type to the same bucket. We conducted a second and sometimes third swipe to capture any potential fish left in the pool. Additionally, we captured fish from a pool located upstream from each site to be later used in the increased density treatment for that particular site (see *Treatment composition, release, and recapture*). We transported the fish to the laboratory in high‐density polyethylene bottles. Once in the laboratory, we selected the three pools with the highest density (invariably corresponding to the largest pools) among those fished, and randomly assigned them to one of the three density treatments: “control,” “decreased,” and “increased.” All fish larger than 10 mm of standard length were anesthetized using MS‐222. We recorded standard length and mass, and marked them with visible implant elastomer (Northwest Marine Technologies), a small subcutaneous color injection that allows the identification of individuals upon recapture. Fish larger than 13 mm were given a unique individual mark, composed of unique color combinations placed in 2 out of 8 possible locations on the body. Fish between 10 and 13 mm were given a single “cohort” mark that was pool, microhabitat, and size‐specific (to the 1‐mm accuracy). This allowed us to make inferences about growth and survival to the nearest mm despite the lack of individual marks. All fish that were visibly smaller than 10 mm length were counted but not processed to avoid overstressing them with anesthesia.

### Treatment composition, release, and recapture

When organizing the treatments, the “control” density pool received the same individuals that were captured there. The “decreased” density treatment was generated by removing a proportion between 40% and 62% of the local population. The “increased” treatment was generated by adding individuals captured upstream from the site into the pool, to generate a final density that was between 128% and 163% of the initial population density. We returned individuals to the same pool they were captured in to avoid confusing dispersal with homing behavior (with the exception of the fish used to augment the “increased” treatment). We kept the sex ratio and size structure proportional to the initial ones, both when increasing and decreasing density. This was done by grouping individuals in size classes of increments of 2 mm and adding or removing fish to/from each group independently. When this was not possible, we removed or added fish to the nearest class above or below it. We released guppies according to the treatment they belonged to. We released guppies removed from the decreased density pool some distance below the bottom barrier waterfall (or in the case of the upstream section of Caigual, downstream from the contiguous site). We fished all three sites again after a period of 20–38 days (depending on the site), adopting the same technique used during the capture. We fished the entire section, including both manipulated and unmanipulated pools, in order to capture dispersed individuals. If an individual was marked, we measured it again to estimate growth and change in body mass. We also measured and weighed individuals without a mark (down to 10 mm). We euthanized approximately 20% of the individuals with an MS‐222 solution and preserved them in formalin for gut content analyses (not analyzed here). All remaining fish were returned to the same pool they were captured in.

### Habitat measurements

We took spatially explicit measurements of the hydrological properties of each site twice: after the first capture and before the final release. We took measures at multiple grid points along the section, spanning its length and width at intervals of 50–100 cm, making sure to uniformly sample each microhabitat present in a pool. At each grid point, we measured water depth, water velocity using a flowmeter (FP111 Global Water Flow Probe), and scored substrate type (silt, sand, gravel, pebbles and rock). Substrate information was used to produce a substrate score that indicated average coarseness for each habitat. Each substrate was assigned a value of ascending coarseness (silt = 0, sand = 1, gravel = 2, pebbles = 3, rock = 4). Mixed substrate was scored as the average of the substrate values (e.g., sand and gravel = 1.5). We also recorded the presence/absence of fine organic detritus and leaf litter on the bottom. Each grid point was also assigned to the microhabitat type it belonged to by referencing the sketches taken during the capture event.

### Life‐history traits and individual measurements

#### Survival

The technique we used to capture guppies grants a high and stable capture rate (~90%, Reznick et al., 2020), which limits the probability of missing an individual present in a given pool. Therefore, we used recapture as a proxy for survival. Because we surveyed all pools between the two barrier waterfalls and capture probability is high, most individuals missed on recapture were likely to be dead.

#### Recruitment

We estimated the average per capita recruitment at the pool level at a given time as the ratio between the number of new recruits (individuals smaller than 10 mm) and the number of larger individuals (>10 mm) that were present in the pool a month earlier (which approximates the time of birth of new recruits). At the time of recapture, after the manipulation, the average per capita recruitment thus corresponded to the number of new recruits captured divided by the number of individuals larger than 10 mm released in the pool when the manipulation started (20–38 days before). At the time of capture this instead corresponded to the ratio of new recruits captured to the number of captured individuals larger than 10 mm: assuming population size and size structure are at the equilibrium and stable in the site before our manipulation, the number of individuals larger than 10 mm captured prior to the manipulation is a close approximation of the number of individuals of the same size class present in the same pool a month before.

#### Growth and body condition

Individual growth was calculated for each released individual that was recaptured at the end of the manipulation and corresponded to the difference in standard length measured before and after the manipulation. To account for differences in the time between the two measures, we standardized all measures of growth as growth in mm over 30 days. We calculated initial and final body condition (before and after the manipulation) using the Fulton's condition factor, which is the ratio of mass (in milligrams) and the cube of standard length (in millimeters). We then calculated the proportional change in condition over 30 days as:

$$\frac{\left(c_{\text{final}}-c_{\text{initial}}\right)}{c_{\text{initial}}}\times \frac{30}{\text{interval}}$$

The interval corresponds to the number of days between the two calculations of condition that were taken (i.e., the number of days the manipulation lasted in each site); *c*_initial_ and *c*_final_ represent the condition calculated before and after the manipulation, respectively. Individuals measuring below 13 mm of standard length could not be given unique identifier marks (due to size limitations). Since individuals were given a “cohort” mark (described in *Capture and marking*), identifying them to the nearest 1 mm, we estimated growth as the difference between the size at recapture and the average size of all individuals belonging to a cohort at capture.

#### Dispersal and microhabitat use

We considered an individual to have dispersed if it was found in a different pool to that it was released in. In addition, we were able to track changes in microhabitat use for individuals that did not disperse due to our spatially explicit capture‐recapture design. For individuals between 10 and 13 mm, the pool‐ and microhabitat‐specific “cohort” mark they received allowed us to infer whether they dispersed to a different pool and, if they did not disperse, whether they moved to a different microhabitat within the same pool. Given that individuals below 10 mm were not marked, we were able to observe dispersal and microhabitat use only for individuals larger than 10 mm.

#### Sex

Individuals were assigned to one of three sex categories: immature, mature male, and mature females. Females smaller than 14 mm were considered immature (Reznick & Endler, 1982), whereas we determined male sexual maturity based on the morphology of the gonopodium (Turner, 1941).

### Statistical analyses

To analyze the probabilities of survival, dispersal, and microhabitat shift, we fit a generalized linear mixed model (GLMM) with binomial error distribution and a logit‐link function, and included site as a random factor and density treatment, size, and sex as explanatory variables together with all two‐ and three‐way interactions among them. We included sex to account for males' shorter life span and high propensity to disperse. The analyses only accounted for the individuals that were present in a given pool before the manipulation, therefore excluding newly introduced individuals into the increased density treatment. We did this to avoid confounding the effect of density with the effect of translocation (e.g., homing behavior).

To analyze in more detail microhabitat choice among the five types, we used a discrete‐choice model (DCM) fitted on all individuals (even those introduced in the increased density treatment). These models are commonly used in econometrics and social sciences, and have been adapted to ecological work (Bonnot et al., 2011; Carter et al., 2010; Cooper & Millspaugh, 1999; McCracken et al., 2006; McDonald et al., 2006; Vardakis et al., 2015). DCMs are a form of regression that model multinomial choices among varying sets of alternatives and allow one to include covariates that vary at the individual level, at the alternative level, and at the choice situation level. For these reasons they are well suited to our study. We included relative benthic area, defined as the proportion of pool occupied by a specific microhabitat type, as an alternative‐specific covariate, while standard length (which was set to 9 mm for all unmeasured individuals smaller than 10 mm), sex, and density treatment and all two and three‐way interactions among them were included as individual‐level covariates. We fit the DCM model with a multinomial logit‐link function.

To analyze growth and body condition difference we fit a linear mixed model (LMM) that included site identity as a random factor, and initial standard length, density treatment, dispersal and all their two‐ and three‐way interactions as fixed effects. The model for growth included a random factor defining the pair of authors who measured the standard length at capture and recapture, to account for potential biases in the manual measurement of length. We note that regressing growth on initial length corresponds to assuming a von Bertalanffy growth curve (where growth is linearly and negatively correlated with initial size), which has been shown to be an adequate growth model in guppies (Bassar et al., 2013; Reznick et al., 2012). We coded dispersal as a binary variable set to 1 when the individual had dispersed to a different habitat patch and to 0 otherwise. Such a variable allowed us to distinguish the effects of density on the growth of individuals who dispersed from the habitat patch and those who remained there, while also accounting for size‐specific responses. We then fit the models again on a subset of the data which included only individuals who did not disperse, and included as a covariate a binary factor indicating whether individuals were found in the same microhabitat type as at capture (0) or if they moved to a different microhabitat type within the same pool (1). This model allowed us to test the correlation between microhabitat shift and performance (growth and body condition). Again, we excluded individuals who were introduced in the increased density treatment. We analyzed average per capita recruitment at the pool level using a linear model where density treatment was the only covariate.

For the analyses of dispersal, survival, growth, and condition, we adopted the following model selection approach. Each model was fitted twice, once using density as a categorical value (“control” vs. “increased” vs. “decreased”) and once as a continuous value (corresponding to the proportional change in density, e.g., 1.50 for a 50% increase in population density, and 0.50 for a decrease of 50%). The percentage by which density was increased or decreased in each site differs, motivating the inclusion of density treatment as a continuous value. While a continuous treatment of density is also more parsimonious, it does not allow for the effects of density to be nonlinear and asymmetric, as found before (Bassar et al., 2013; Reznick et al., 2012). For this reason, we tested both types of models. We then simplified each of the two models (with density as categorical or continuous value) with a step‐down approach, starting from a full model with all interactions among factors and removing hierarchically any term that was not significant at the α = 0.05 level (*Z* test for binomial, *t* test for normal distribution). Finally, we compared the Akaike information criterion (AIC) values of the two resulting models. When the difference in AIC between models (ΔAIC) was below 4, we considered the most complex of the two models, and picked the model with the lower AIC value otherwise. The DCM and the GLMM to test for microhabitat shift could not reach convergence when fitted using density treatment as a categorical value, so we fitted them only using density as a continuous variable and proceeded to simplify the model as explained above. To test for nonlinear effects of density, we included a quadratic term for density in the microhabitat shift model. We did this because we expected individuals could be likely to change microhabitat in both increased and decreased density treatments, moving to a better or worse microhabitat, respectively. The model for average per capita recruitment needed no simplifying procedure since it included only one covariate.

In all models, standard length was scaled and centered to improve model convergence. We conducted all analyses in R (version 3.5.1; R Development Core Team, 2008), fitting GLMM and LMM using the functions *glmer* and *lmer* (package lme4; Bates et al., 2015) and the DCM using the function *mlogit* (package mlogit; Croissant, 2020).

## RESULTS

### Survival and recruitment

**FIGURE 1:**
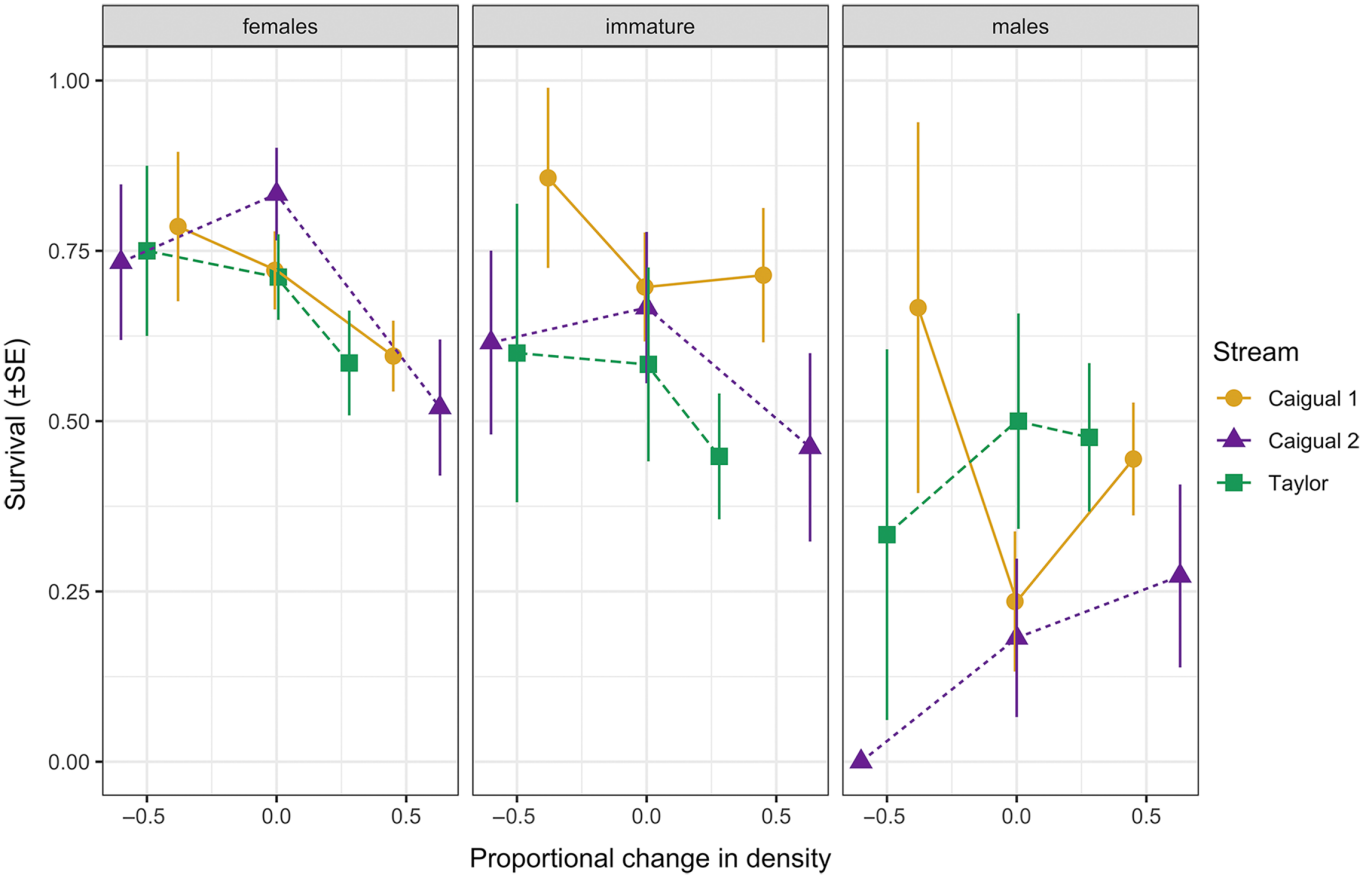
Effect of density treatment on the average survival probability (mean ± SE) in the three experimental streams, considered separately for females, males, and immature individuals (regardless of sex). Shown are the average individual survival probabilities for the three study sites: the upper range of Caigual (Caigual 1), the lower range of Caigual (Caigual 2), and Taylor.

Two models tied for best fit for survival, and both included covariates for density treatment, size, sex, the interaction between size and sex, and the interaction between density treatment and sex (Appendix S2: Table S1). The models differed in the way density treatments were treated, either as a continuous variable (AIC = 781.23) or as a categorical variable (AIC = 783.26). The results are consistent between the two models, so we will here present the former and describe the latter in Appendix S4: Table S1. Larger females had lower estimated survival than smaller ones, and overall females of any size had higher survival than both juveniles and males of the same size. High density decreased survival in females (Appendix S3: Table S1). An average‐sized female is predicted to have approximately 87% survival rate when density is reduced by 50%, compared to the 59% survival rate when density is increased by the same percentage (Figure 1). For juveniles in all sites, survival was higher in the decreased density treatment compared to the increased density treatment (Figure 1). Males show relatively higher survival when at high density.

**FIGURE 2:**
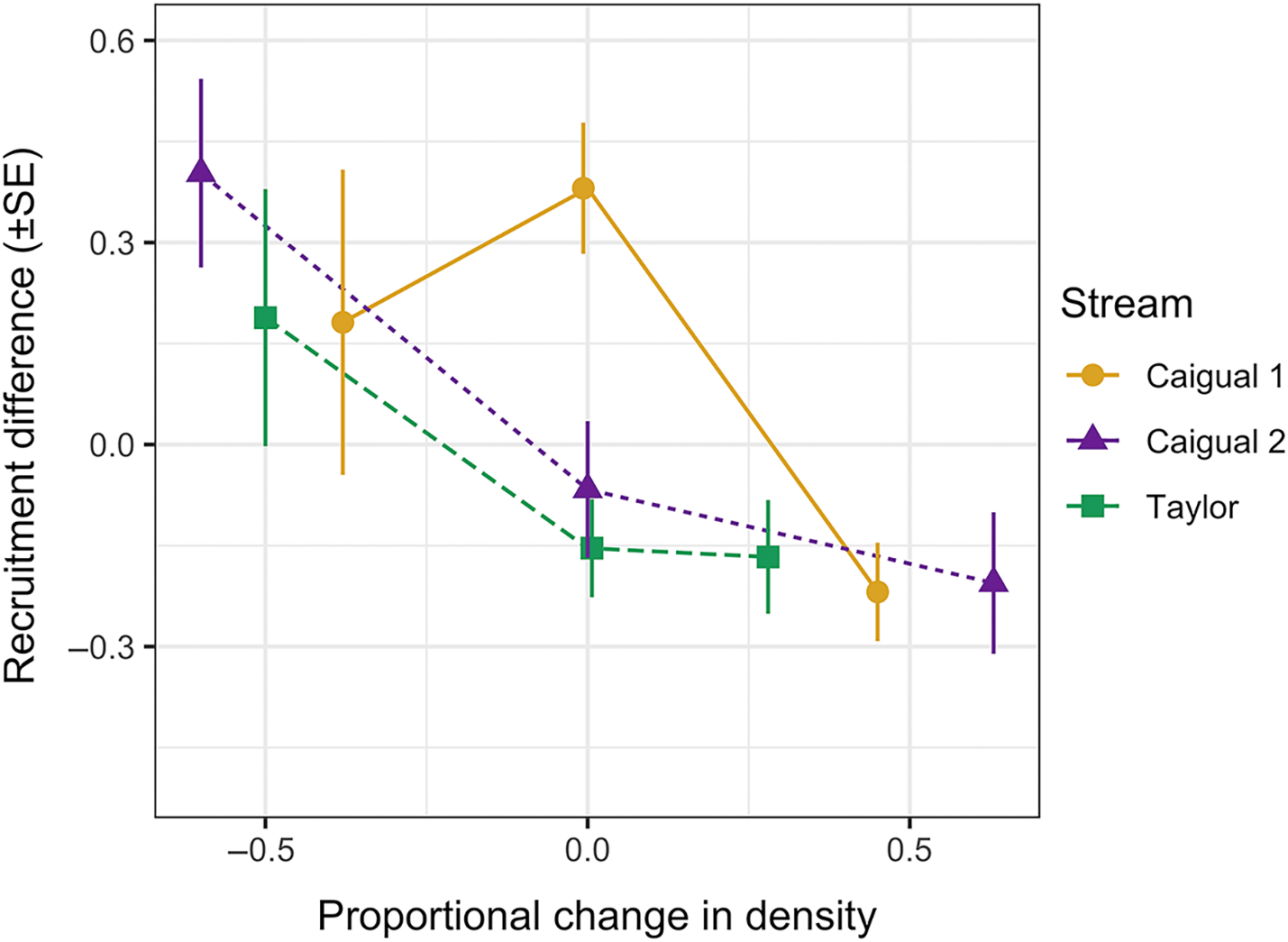
Effect of density treatment (expressed as the proportional relative change in density) on the difference in estimated per capita recruitment (mean ± SE), calculated at the pool level, between first capture and recapture in the three study sites: the upper range of Caigual (Caigual 1), the lower range of Caigual (Caigual 2), and Taylor. SE is calculated using the delta approximation, assuming a Poisson error distribution for the count data, and no covariance between recruitments in first capture and recapture.

Average per capita recruitment at the pool level decreases significantly with density (GLMM effect size = − 0.472 ± 0.138, *p* = 0.011, Figure 2).

### Dispersal

**FIGURE 3:**
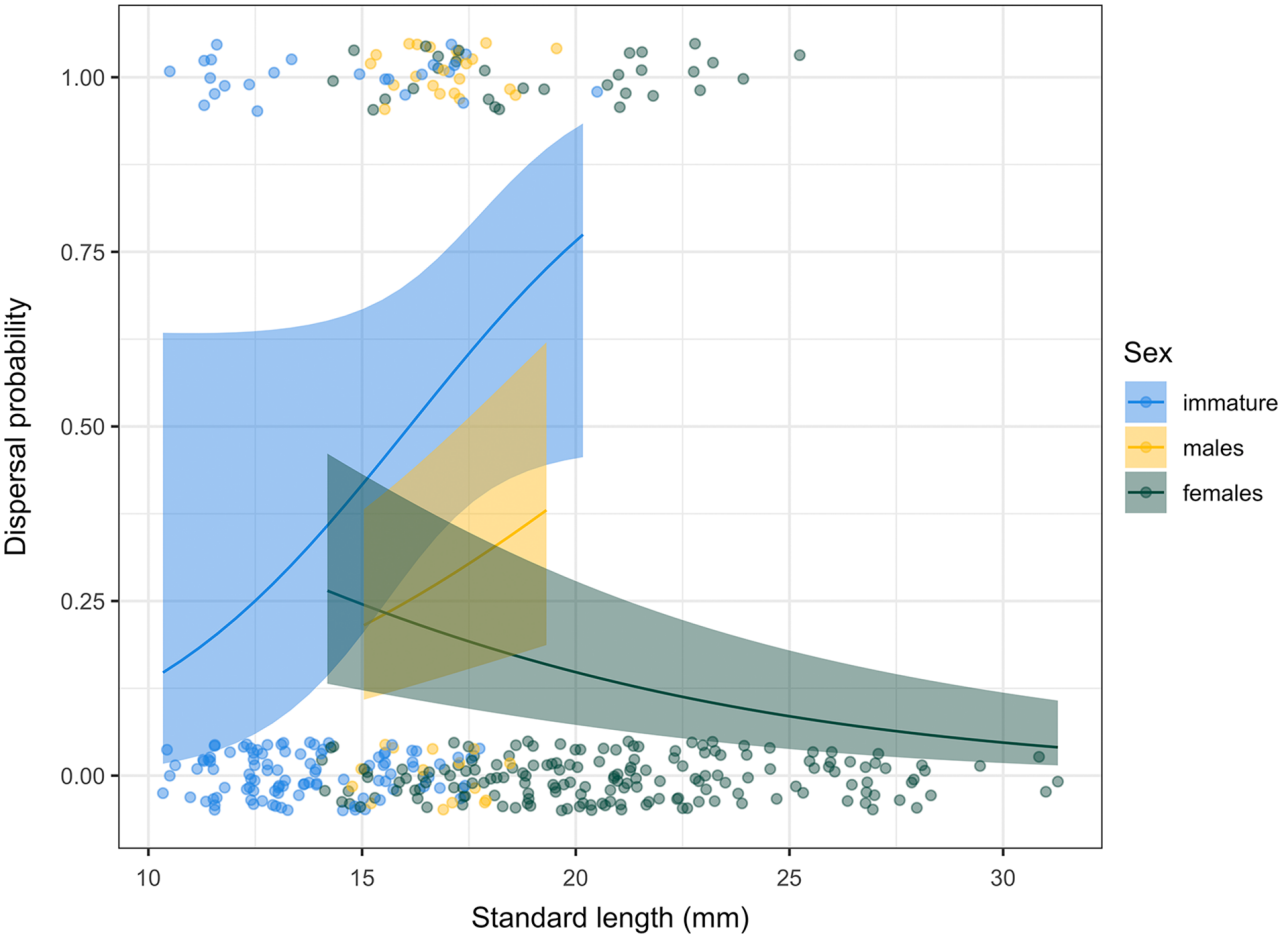
Dispersal probability based on size (standard length) and sex: males, females and immature individuals (regardless of sex). Lines represent the dispersal probability predicted by the generalized linear mixed model (GLMM); shaded areas represent the SE on those predictions (marginalized over the random effect); circles represent the observed dispersal events and can take values of 0 if an individual did not disperse or 1 if it did. The points are jittered over the *y*‐axis to reduce overlap.

The best fit model for dispersal probability included the main effects of size, sex and their interaction, and did not include density treatment (Appendix S2: Table S2). Males were overall more likely to disperse than females. Large females were less likely to disperse than smaller ones (Figure 3; Appendix S3: Table S2).

### Microhabitat characteristics and microhabitat use

The mean values for depth, flow, substrate score, and the probability to find detritus and leaves in all microhabitats are summarized in Table 1. Mean depth was highest in the core, inflow, and run, whereas water depth in marginal microhabitats such as swamps and beach was approximately half to a third. As expected, water velocity was higher in the narrower inflow and outflow of the pools, and resulted in less deposition of organic matter. The average substrate was coarsest in these two microhabitats as well (2.63–2.70), and finest in marginal habitats (1.42–1.80).

**TABLE 1:** Summary of microhabitat features.

Microhabitat	Depth (cm)	Flow (m/s)	Substrate (score)	Detritus (*p*)	Leaves
Inflow	11.84 [1.85–37.2]	0.32 [0–1.0]	2.71 [1–4]	0.04	0.05
Beach	4.9 [0.97–13.53]	0.03 [0–0.2]	1.42 [0.49–4]	0.44	0.18
Core	15.11 [1–43.6]	0.09 [0–0.4]	2.27 [1–4]	0.55	0.25
Swamp	6.37 [0–17.3]	0.01 [0–0.1]	1.80 [0–4]	0.75	0.59
Run	10.27 [0.58–22]	0.20 [0–0.7]	2.63 [1–4]	0.24	0.12

*Note*: Intervals represent 95% quantiles and are omitted for detritus and leaves (as both are binary variables defining presence or absence of detritus and leaf litter, scored as 1 and 0, respectively).

**FIGURE 4:**
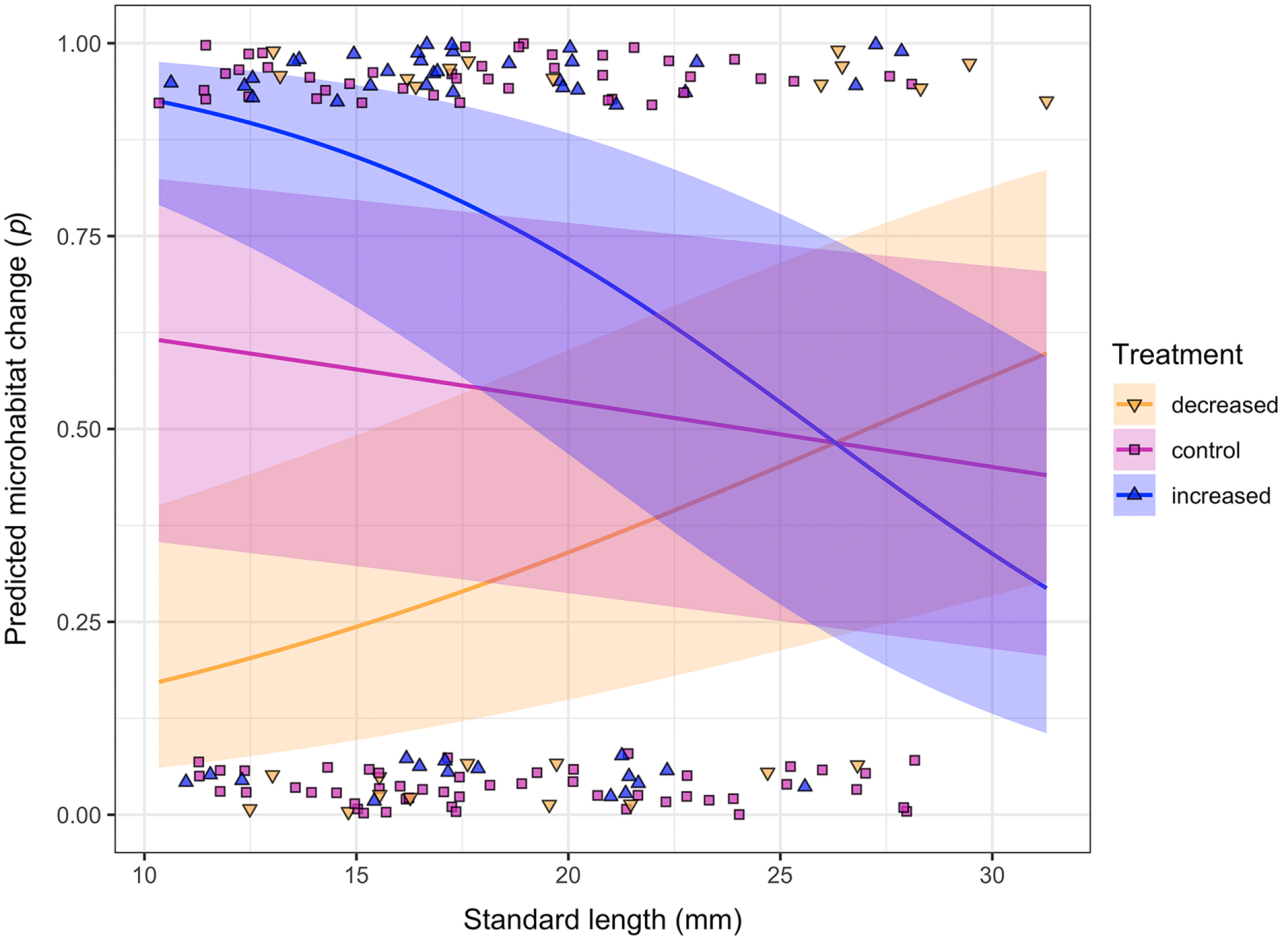
Predicted probability to shift between microhabitats based on density and size (standard length), for individuals who did not disperse. Lines represent the probability estimated with the generalized linear mixed model (GLMM), with SE for the predictions (marginalized over the random effect) depicted as shaded areas, for control (unmodified, blue), decreased density (by 50%, yellow), and increased density (by 50%, pink). Each symbol represents the observed microhabitat shift of an individual in the different density treatments: decreased density, increased density, and control. The observed values are 0 (no microhabitat shift) or 1 (shift in microhabitat) and are jittered over the *y*‐axis to reduce overlap.

First, we tested if density manipulation caused non‐dispersing individuals to alter their microhabitat use. The best fit model included size, density (expressed as a continuous variable), and their interaction (Appendix S2: Table S3). The effects of density and its interaction with size were both significant (Table 2). At decreased density, large individuals were more likely than small ones to shift microhabitat. On the other hand, at both control and increased density, large individuals were more likely to remain in the same microhabitat while small individuals were likely to shift (Figure 4).

**TABLE 2:** Generalized linear mixed model for guppy microhabitat shift as a function of size and density treatment (*N* = 285).

Parameter	Estimate	SE	*Z* value	*p* value
(Intercept)	0.211	1.049	0.201	0.841
Size (mm)	−0.153	0.126	−1.223	0.222
Density	2.133	0.504	4.235	<0.001***
Size × Density	−1.157	0.401	−2.882	0.004**

*Note*: **p < 0.01, ***p < 0.001.

Second, to explore the direction of microhabitat change, we fitted a DCM. The best DCM includes the effect of size, sex, and density treatment (as a continuous variable) and all two‐and three‐way interactions among them, together with the alternative‐specific covariate for relative benthic area occupied by each microhabitat in the pool. The results of the DCM refer to the effects of each covariate (or interaction between covariates) on the odd ratio to choose each microhabitat over the inflow, our reference level (Appendix S5: Table S1). The coefficients are used to generate the predicted probability of occupying a microhabitat based on size, sex, and density treatment (Appendix S5: Figure S1). Here, we analyze the results visually and qualitatively, exploring the predicted probabilities of occupying microhabitats in Appendix S5: Figure S1 and referring to Appendix S2: Table S2 for more detailed comparisons. The estimated coefficients for microhabitat use in males display high uncertainties (Appendix S5: Table S1) making it difficult to pinpoint their microhabitat use with confidence; we will not discuss them here. At control densities, females occupy inflow and core microhabitats disproportionately, and as their size increases, the probability of occupying the inflow increases at the expense of all other microhabitats. Compared to females, immature individuals are more likely to occupy marginal areas such as beach and swamp, especially when small, although a large proportion of medium‐sized immature individuals occupy the core. Large immature individuals become more likely to occupy inflow and run rather than beach, swamp, and core. Density does not affect microhabitat use in females significantly, although for large females, the occupancy of inflow increases and that of core decreases when density is increased, and vice versa when density is decreased. Immature individuals become more likely to occupy swamp (if small), run, and core (if medium‐sized or large) when density increases. Large immature individuals occupy the inflow disproportionately when density decreases.

### Effect of dispersal on growth and change in body condition

**FIGURE 5:**
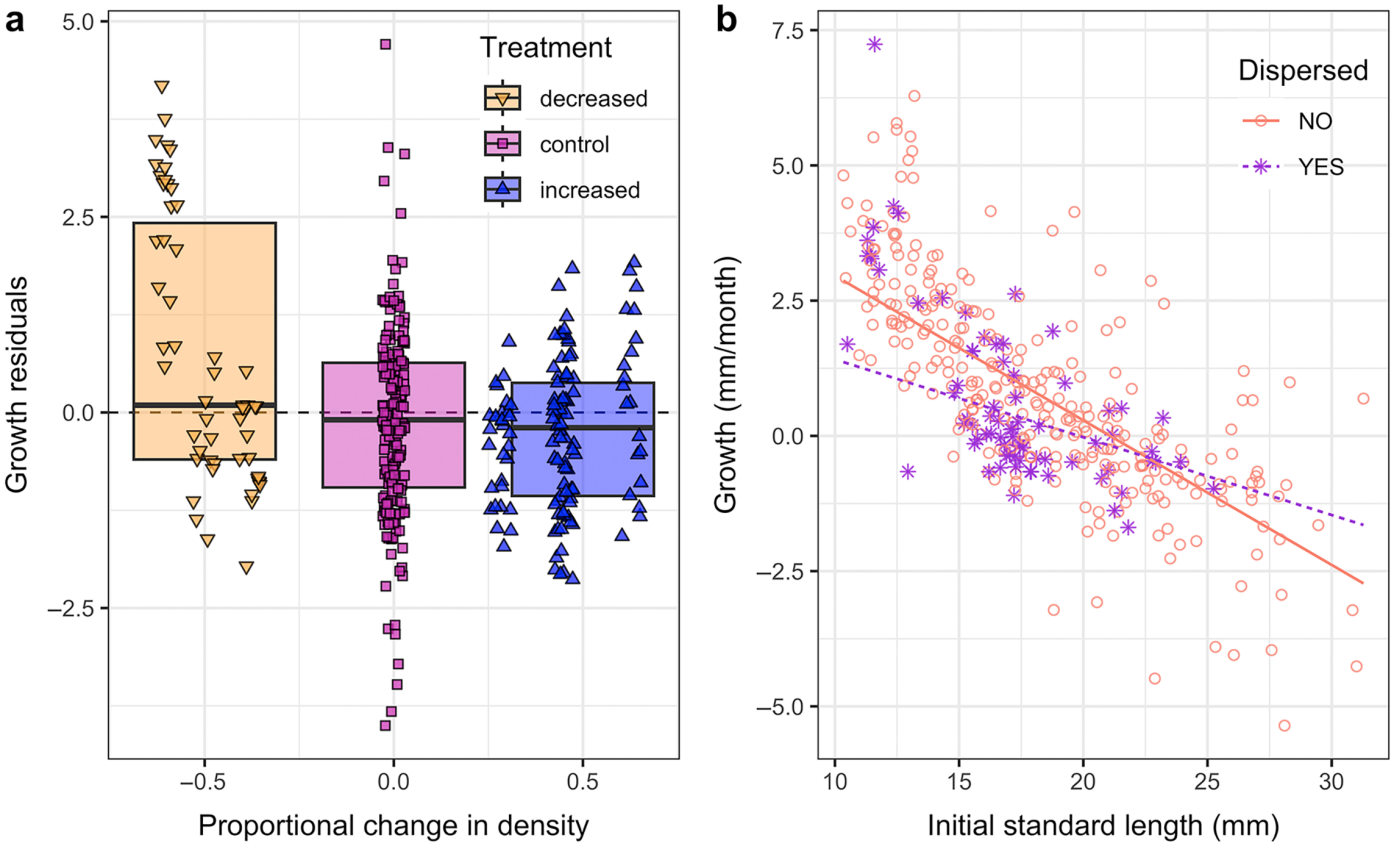
(a) Size‐specific residual growth in different density treatment levels, expressed as proportional change in density. Yellow downward‐pointing triangles represent decreased density; pink squares represent control density; blue upward‐pointing triangles represent increased density. The points are jittered over the *x*‐axis to reduce overlap. The shaded boxes represent interquartile ranges from the data, pooled by treatment (decreased, control and increased). (b) Lines represent the predicted size‐specific growth at control density for individuals that remained in the same pool (solid) or dispersed (dashed). Density treatment does not interact with size or dispersal; therefore, the slope of the predicted curves is the same, despite the intercept being different. Points represent observed growth values for individuals that remained (circles) or dispersed (asterisks).

The best fit model for growth included density treatment as a continuous variable, as well as size, dispersal (yes/no), and their interaction (Appendix S2: Table S4). Higher density decreased individual growth for all individuals equally, regardless of size and dispersal. Growth was negatively correlated with initial size: larger individuals grew less than smaller ones. The negative and significant effect of dispersal, together with the positive and significant interaction between size and dispersal, suggests a size‐specific cost of dispersal: small individuals grew less when dispersing compared to similarly sized individuals that remained in the same pool (Figure 5; Appendix S3: Table S3). Large individuals, on the other hand, grew relatively more if dispersing compared to same‐sized individuals remaining in the same pool.

**FIGURE 6:**
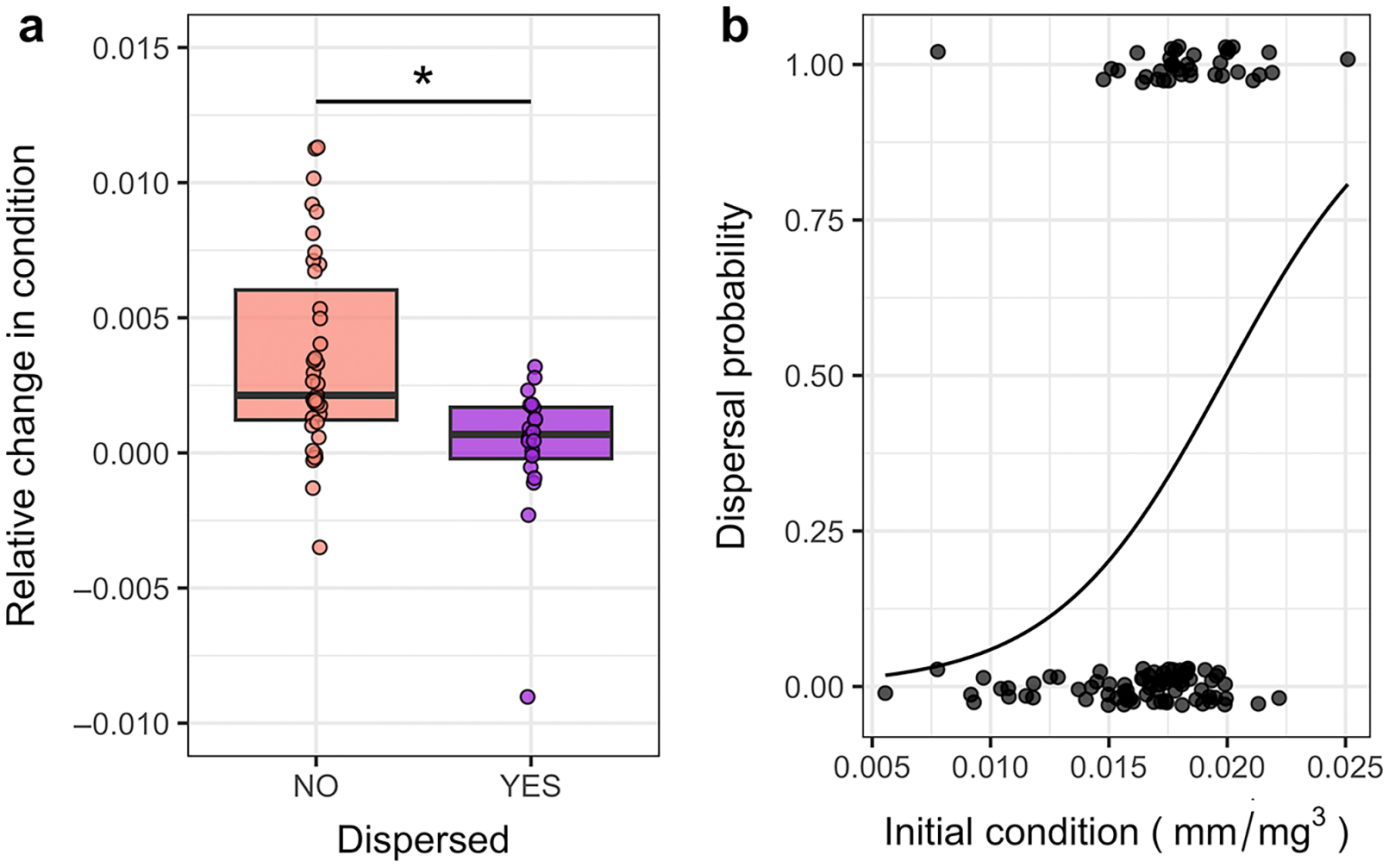
(a) Effect of dispersal on the relative change in body condition over 30 days. Points represent the data: pink for individuals who did not disperse; purple for individuals who did disperse. Shaded boxes represent interquartile ranges from the same data. The asterisk represents a significant difference at α = 0.05. (b) Effect of initial condition on the probability to disperse. The solid line represents the estimate from the generalized linear mixed model (GLMM); points correspond to the observed dispersal events by individuals of varying conditions, jittered over the *y*‐axis to reduce overlap.

The best fit model for the proportional change in condition over 30 days only included the effect of dispersal, thus density treatment had no effect on body condition (Appendix S2: Table S5). Across all sizes, the condition of individuals that remained in a patch improved more than that of those who dispersed (LMM: effect size = −0.002 ± 0.001, *p* < 0.001; Figure 6a). To explore the direction of the causal relationship between condition and dispersal, we ran two additional models. Individual condition before the experiment positively affected the propensity to disperse, meaning individuals in good condition were more likely to abandon the patch (GLMM: effect size = 0.277 ± 0.095, *p* = 0.004; Figure 6b). In addition, dispersal negatively affected individual condition after the experiment (LMM: effect size = 9.452 ± 4.304, *p* = 0.032).

### Effects of microhabitat shift on growth and condition

**FIGURE 7:**
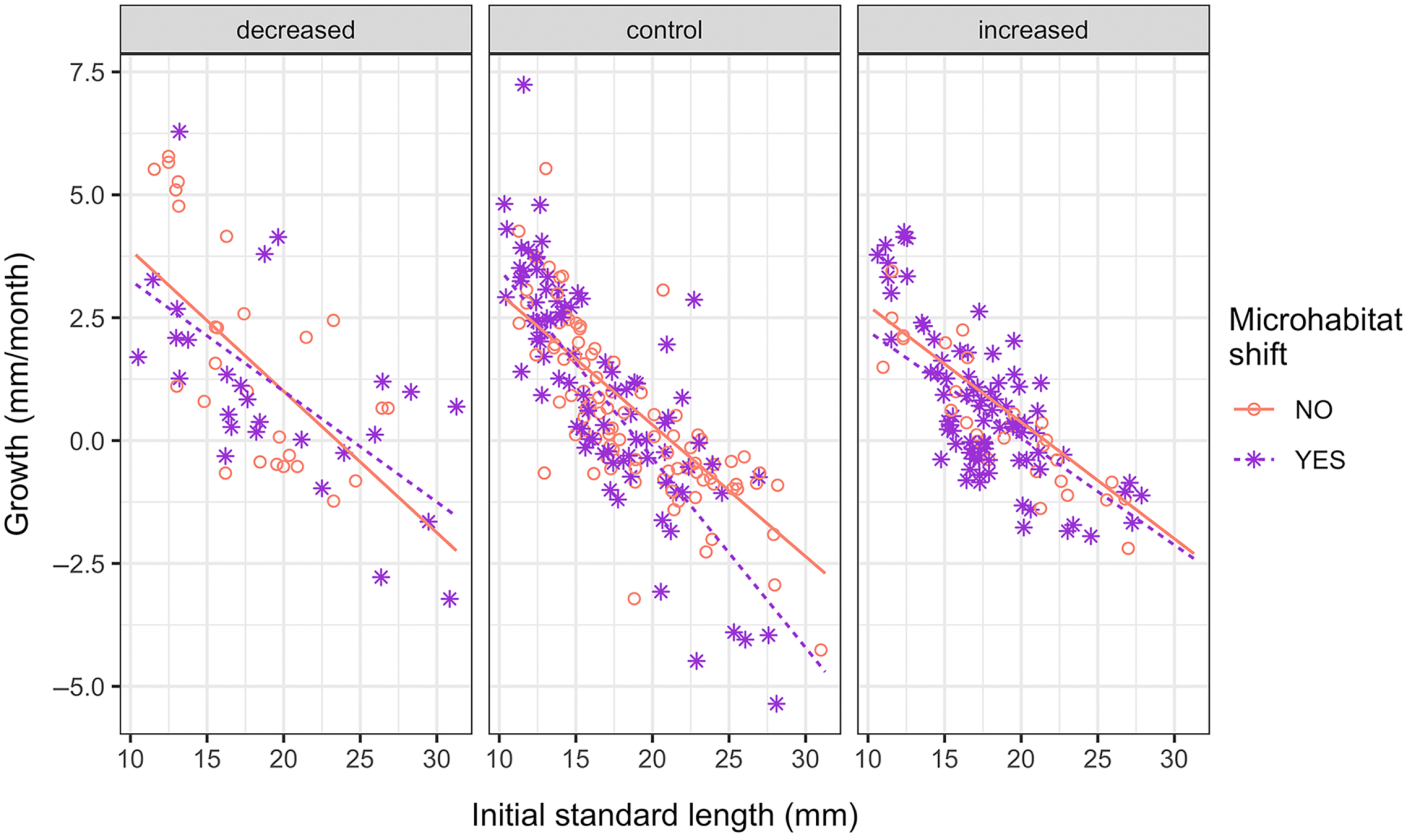
Size‐specific effect of habitat shift and density on growth, for individuals who did not disperse. The lines represent the estimated size‐specific growth for individuals that changed microhabitat type (purple, dashed) or did not (pink, solid). The points represent observed individuals that shifted microhabitat (purple asterisks) or did not (pink circles).

When considering only the individuals who did not disperse and how microhabitat change affected their growth, two models tied for best (Appendix S2: Table S6). The best fit model including density treatment as a categorical factor also included initial size, microhabitat shift (yes/no), and all two‐ and three‐way interactions among them. The model selection for the LMM including density as a continuous variable led to dropping microhabitat shift from the covariates. Fitting density as a categorical variable can highlight responses to density that are not captured by a simpler linear relationship and would be lost in a model that includes density as a continuous variable. Therefore, here we consider the model where density treatment is expressed as a categorical variable (Appendix S3: Table S4). In the control treatment, large individuals that did not shift to a different microhabitat grew less compared to small ones, as expected. Shifting to a different microhabitat decreased the growth of large individuals, suggesting they might hold good quality microhabitats at equilibrium density. Average‐sized individuals have significantly higher growth in the decreased treatment if they remain in the same habitat. Large individuals in the decreased treatment benefit significantly from shifting their habitat, whereas the growth of small individuals is improved when remaining in the same habitat at decreased density (Figure 7; Appendix S3: Table S4).

The best model of the difference in condition among individuals that did not disperse includes standard length and microhabitat shift (no/yes) (Appendix S2: Table S7). The effect of microhabitat shift was not significant (LMM: SL effect size = −0.0034 ± 0.0015, *p* = 0.0303; habitat change effect size = 0.0006 ± 0.0009, *p* = 0.5631), but the sample size was very small (*N* = 39).

## DISCUSSION

We manipulated the density of guppies in natural habitat patches to observe its effect on vital rates, and on the spatial responses between and within patches (dispersal and microhabitat shift, respectively). In density‐regulated populations, density perturbations are expected to affect demographic traits and regulate the population back to its equilibrium (Egerton, 1973; Nicholson, 1933; Turchin, 1995). In our study, increased density decreased the survival of females and immature individuals (Figure 1), and the growth of all individuals (Figure 5a). Moreover, the number of per capita new recruits at the pool level was decreased by high density and increased by low density (Figure 2). This corroborates previous density manipulation experiments in various species (Matthews et al., 2001; Petranka, 1989; Rodenhouse et al., 2003; Tanaka et al., 1999; Wilbur, 1977).

Previous manipulations in wild guppy populations have shown similar regulation patterns, with decreased density causing increased survival, growth, and reproductive allocation, and increased density reducing fat reserves (Bassar et al., 2013; Reznick et al., 2012), although some key differences emerge. While size and density treatment do not interact, in our case, Reznick et al. (2012) found asymmetries in life history responses to density based on size. For instance, when density was increased, survival was further decreased in large guppies than small ones. The decrease in growth, on the other hand, was more severe in small individuals than large ones. The ability to detect these asymmetries might be due to the larger sampling effort involved in this previous study. While Reznick et al. (2012) did not test for sex differences, we found males to have higher survival at high density, a counterintuitive result. This unexpected result may be due to the high dispersal propensity observed in males (Figure 3). Dispersing early after the manipulation could cause pool density and survival to become unlinked.

Despite the impact of density on vital rates, we did not observe a direct effect of increased density on dispersal. Positive density‐dependent dispersal, whereby individuals leave crowded patches to escape strong conspecific competition for resources, is widespread (Bitume et al., 2013; Doak, 2000; Einum et al., 2006; Léna et al., 1998; Matthysen, 2005) and previous observational studies show that guppies are more likely to leave a patch if local density is high (De Bona et al., 2019). We noted a high variation in dispersal behavior among our replicates and in different density treatments: in pools where density was decreased we recorded both the highest and lowest dispersal rates (respectively 0.47, and no dispersers), whereas the three control pools showed very consistent proportions of dispersers (0.12–0.23). Pools where density was artificially decreased have small sample sizes by experimental design, and can therefore be more subject to stochastic fluctuations. Dispersal is a behavior with many proximate drivers (Matthysen, 2012), and short‐term manipulation studies like ours might fail to detect density effects (Clutton‐Brock et al., 1985; Massot et al., 1992).

Estimating the cost of dispersal may offer more insight into why individuals did not escape from increased density treatments. Our results show dispersal negatively affects body condition, regardless of size and density treatment (Figure 6a). This could indicate that individuals with previously low body condition have a higher likelihood of dispersal, but our analyses show the opposite, with individuals in good condition more likely to disperse (Figure 6b), as shown in previous studies (Debeffe et al., 2012; Delgado et al., 2010; Meylan et al., 2002). Moreover, we found dispersal to disproportionately impact the growth of smaller size classes (Figure 5b). Costs of dispersal for mobile individuals can be linked to the energetic demands of prolonged movement (Bowlin et al., 2005; Fish et al., 2001; Roff, 1977; Srygley & Ellington, 1999), but evidence of the consequences of dispersal on body condition is scarce.

At smaller spatial scales (within patch), we found microhabitat use to be affected by size and sex/stage. At equilibrium density, females preferred central areas of the pool (core) and microhabitats with fast‐running water (inflow and run), while immature individuals were more evenly distributed among microhabitats, and found in relatively high proportions in marginal microhabitats (“beach” and “swamp”) (Appendix S5: Figure S1). The inflow microhabitat requires constant swimming against fast‐running currents—which might be sustainable only for large individuals—but can provide opportunities for drift feeding on invertebrates (S. De Bona, personal observation). The microhabitats most occupied by large (>15 mm) individuals were “core” and “inflow,” which corresponded to the deepest areas of a pool (Table 1). This aligns with the size segregation relative to water depth found in previous studies (Croft et al., 2003), and can cause microhabitat segregation among the sexes (Croft et al., 2004; Darden & Croft, 2008).

The observation of small‐scale behaviors allowed us to evaluate the effects of density on microhabitat use. We found microhabitat shift to be density and size/stage dependent (Figure 4; Appendix S3: Table S3). We argue that this pattern, in combination with the observed effects of microhabitat shift on individual growth (Figure 7; Appendix [Link], [Link]: Table S4), can be explained by dominance interactions. Previous behavioral assays in controlled conditions showed that dominant individuals tend to be the larger ones in a group (Borg et al., 2012; Gorlick, 1976; Magurran & Seghers, 1991). In our study, when density is increased, large individuals are more likely to remain in the microhabitat they were inhabiting, while small individuals are likely to move. The opposite is true in decreased density conditions (Figure 4). At increased density, the growth of all individuals is negatively affected by microhabitat shift (Figure 7). The higher probability of microhabitat shift by small individuals might be the consequence larger individuals displacing them. When density is decreased and competition relaxed, microhabitat shift is beneficial for large individuals (which move more often) and detrimental to small ones (which instead tend to remain in the same microhabitat). Displacement of small individuals by larger ones is documented in other fish species (see Davey et al., 2005). It is important to note that size and age are tightly linked in guppies, especially in females, which display indeterminate growth. For this reason, we are unable to disentangle the effect of size per se and that of age and experience. A longer term experiment, where individuals are followed throughout their life span, would help clarify the separate role of size and experience.

Dominance interactions potentially driving the pattern of microhabitat shift suggest that guppies follow an ideal despotic, rather than free distribution, at least at the small (within patch) scale. A long‐term study following introduced guppy populations during the establishment phase showed guppies were more likely to move from pools to riffles (a less preferred habitat) as population density increased (Reznick et al., 2020). Despite being suboptimal habitats, riffles are characterized by much lower population density compared to pools, so the lower competition equalized fitness (lifetime reproductive success and lifetime mating success) between the two habitats. This pattern suggests at larger scales (between patch), guppies might conform to an ideal free distribution. There is experimental evidence for both ideal despotic (Haugen et al., 2006) and ideal free distribution in fish (Church & Grant, 2019; Purchase & Hutchings, 2008). Our results, combined with previous research, suggest different processes might be at play even in the same species, depending on the spatial scale considered (Bult et al., 1999).

## AUTHOR CONTRIBUTIONS

Sebastiano De Bona conceived the study. Sebastiano De Bona and Andrés López‐Sepulcre designed the study, with contributions from Karendeep Sidhu and Hanna M. Enroth. Sebastiano De Bona, Karendeep Sidhu, and Hanna M. Enroth conducted the experiment. Sebastiano De Bona performed the analyses and wrote the manuscript with Andrés López‐Sepulcre. All authors contributed to the discussion of results and commented on the manuscript.

## CONFLICT OF INTEREST STATEMENT

The authors declare no conflicts of interest.

## Supporting information


Appendix S1.

Appendix S2.

Appendix S3.

Appendix S4.

Appendix S5.

## Data Availability

Data and code (De Bona, 2025) are available in Zenodo at https://doi.org/10.5281/zenodo.15485445.
