# Supplementary material for: Density‐dependent dispersal and habitat use in size‐structured populations: An experiment in wild Trinidadian guppies
Source: Ecology. 2025 Jul 18;106(7):e70151. doi: 10.1002/ecy.70151 (PMC12272143; doi:10.1002/ecy.70151)
Supplement: Supplementary file 1 — Appendix S1. [file ECY-106-e70151-s003.pdf]

Density-dependent dispersal and habitat use in size-structured populations: An experiment in wild Trinidadian guppies

Sebastiano De Bona, Karendeep Sidhu, Hanna M. Enroth & Andrés López-Sepulcre

Ecology

Appendix S5 – Population sizes and densities

Table S1. Numerical values of population size and density in all experimental pools.

Stream	Pool treatment	Pop. size	Pop. density (1/m2)
Caigual I	Control	193	7.81
Caigual I	Decreased	42	2.91
Caigual I	Increased	163	10.19
Caigual II	Control	106	8.46
Caigual II	Decreased	60	2.08
Caigual II	Increased	49	6.60
Taylor	Control	100	8.30
Taylor	Decreased	28	5.54
Taylor	Increased	63	5.14
