# Supplementary material for: Density‐dependent dispersal and habitat use in size‐structured populations: An experiment in wild Trinidadian guppies
Source: Ecology. 2025 Jul 18;106(7):e70151. doi: 10.1002/ecy.70151 (PMC12272143; doi:10.1002/ecy.70151)
Supplement: Supplementary file 2 — Appendix S2. [file ECY-106-e70151-s005.pdf]

Density-dependent dispersal and habitat use in size-structured populations: An experiment in wild Trinidadian guppies

Sebastiano De Bona, Karendeep Sidhu, Hanna M. Enroth & Andrés López-Sepulcre

Ecology

Appendix S1 - Model selection

Tables of AIC values used for model comparison. Variables listed: SL = standard length, sex, d = density, disp = dispersal (yes/no), shift = microhabitat shift (yes/no). The colon represents an interaction among factors. The "+" symbol indicates which variables were present in the model. Models selected and described in the main text are in bold.

Table S1. a) Survival probability, with density as a continuous variable.

SL	sex	d	SL:sex	SL:d	sex:d	SL:sex:d	AIC
+	+	+	+	+	+	+	781.94
+	+	+	+	+	+		781.23
**+**	**+**	**+**	**+**		**+**		**780.26**

b) Survival probability, with density as a categorical variable

SL	sex	d	SL:sex	SL:d	sex:d	SL:sex:d	AIC
+	+	+	+	+	+	+	789.37
+	+	+	+	+	+		786.46
+	+	+	+		+		782.90

Table S2. a) Dispersal probability, with density as a continuous variable

SL	sex	d	SL:sex	SL:d	sex:d	SL:sex:d	AIC
+	+	+	+	+	+	+	352.98
+	+	+	+	+	+		353.58
+	+	+	+		+		353.68
+	+	+	+				353.97
**+**	**+**		**+**				**352.08**

b) Dispersal probability, with density as a categorical variable

SL	sex	d	SL:sex	SL:d	sex:d	SL:sex:d	AIC
+	+	+	+	+	+	+	357.94
+	+	+	+	+	+		352.97
+	+	+	+		+		355.96
+	+	+	+				354.90
**+**	**+**		**+**				**352.08**

Table S3. Probability of microhabitat shift, with density as a continuous variable.

SL	sex	d	SL:sex	SL:d	sex:d	SL:sex:d	d2	AIC
+	+	+	+	+	+	+	+	369.95
+	+	+	+	+	+		+	369.98
+	+	+	+	+			+	367.29
+	+	+		+			+	367.45
+	+	+		+				367.69
**+**		**+**		**+**				**364.14**

Table S4. a) Growth as a function of dispersal, with density as a continuous variable.

SL	disp	d	SL:disp	SL:d	disp:d	SL:disp:d	AIC
+	+	+	+	+	+	+	964.08
+	+	+	+	+	+		963.62
+	+	+	+		+		960.76
**+**	**+**	**+**	**+**				**961.22**

b) Growth as a function of dispersal, with density as a categorical variable.

SL	disp	d	SL:disp	SL:d	disp:d	SL:disp:d	AIC
+	+	+	+	+	+	+	968.36
+	+	+	+	+	+		966.15

Table S5. a) Changes in body condition as a function of dispersal, with density as a continuous variable.

SL	disp	d	SL:disp	SL:d	disp:d	SL:disp:d	AIC
+	+	+	+	+	+	+	-415.03
+	+	+	+	+	+		-424.43
+	+	+	+	+			-436.26
+	+	+	+				-460.86
+	+	+					-455.58
+	+						-468.65
	**+**						**-479.05**

b) Changes in body condition as a function of dispersal, with density as a categorical variable.

SL	disp	d	SL:disp	SL:d	disp:d	SL:disp:d	AIC
+	+	+	+	+	+	+	-380.57

Table S6. a) Growth as a function of microhabitat shift, for individuals that stayed, with density as a continuous variable

SL	shift	d	SL:shift	SL:d	shift:d	SL:shift:d	AIC
+	+	+	+	+	+	+	787.95
+	+	+	+	+	+		787.40
+	+	+	+	+			785.82
+	+	+	+				782.71
+	+	+					778.90

b) Growth as a function of microhabitat shift, for individuals that stayed, with density as a categorical variable

SL	shift	d	SL:shift	SL:d	shift:d	SL:shift:d	AIC
**+**	**+**	**+**	**+**	**+**	**+**	**+**	**782.18**

Table S7. a) Changes in condition as a function of microhabitat shift, for individuals that stayed, with density as a continuous variable

SL	shift	d	SL:shift	SL:d	shift:d	SL:shift:d	AIC
+	+	+	+	+	+	+	-243.58
+	+	+	+	+	+		-252.57
+	+	+	+		+		-262.04
+	+	+			+		-271.90
+	+	+					-281.23
**+**	**+**						**-293.52**

b) Changes in condition as a function of microhabitat shift, for individuals that stayed, with density as a categorical variable

SL	shift	d	SL:shift	SL:d	shift:d	SL:shift:d	AIC
+	+	+	+	+	+	+	-231.10
