# Supplementary material for: Density‐dependent dispersal and habitat use in size‐structured populations: An experiment in wild Trinidadian guppies
Source: Ecology. 2025 Jul 18;106(7):e70151. doi: 10.1002/ecy.70151 (PMC12272143; doi:10.1002/ecy.70151)
Supplement: Supplementary file 3 — Appendix S3. [file ECY-106-e70151-s002.pdf]

Density-dependent dispersal and habitat use in size-structured populations: An experiment in wild Trinidadian guppies

Sebastiano De Bona, Karendeep Sidhu, Hanna M. Enroth & Andrés López-Sepulcre

Ecology

Appendix S2 – Additional Result Tables

The following tables contain model outputs for some of the analyses reported in the main text.

Table S1. GLMM for survival (N = 611).

		Estimate	SE	Z value	P value	
Intercept		1.074	0.162	6.6496	< 0.001	***
Size (mm)		-0.308	0.126	-2.545	0.014	*
Sex	immature	-0.929	0.379	-2.453	0.014	*
	males	-2.532	0.392	-6.455	< 0.001	***
Density		-1.213	0.386	-3.143	0.002	**
Size × sex	immature	-0.103	0.347	-0.297	0.767	
	males	-1.384	0.647	-2.138	0.033	*
Sex × Density	immature	0.755	0.638	1.183	0.237	
	males	2.289	0.732	3.126	0.002	**

Table S2. GLMM for dispersal probability (N = 362).

		Estimate	SE	Z value	P value	
(Intercept)		-1.493	0.806	-1.853	0.064	.
Size (mm)		-0.567	0.268	-2.115	0.034	*
Sex:	immature	0.748	0.531	1.410	0.159	
	males	2.055	0.583	3.528	< 0.001	***
Size × Sex:	immature	1.419	0.563	2.519	0.012	*
	males	1.943	1.525	1.274	0.202	

Table S3. LMM for the effect of density and dispersal on growth (N = 312).

	Estimate	SE	z-value	p-value	
Intercept	0.834	0.296	2.823	0.039	*
Size	-1.211	0.073	-16.595	< 0.001	***
Dispersed	-0.565	0.151	-3.740	< 0.001	***
Density	-0.937	0.198	-4.727	< 0.001	***
Size × Dispersed	0.560	0.227	2.461	0.014	*

Table S4. LMM for the effect of habitat change, size and density treatment on the growth of individuals that did not disperse (N = 246).

Coefficients	Factor level	Estimate	SE	t-value	P value	
Intercept		0.868	0.355	2.441	0.053	
Size		-1.212	0.128	-9.447	<0.001	***
Density	decreased	0.723	0.304	2.378	0.018	*
	increased	-0.009	0.334	-0.028	0.977	
Microhabitat shift		-0.439	0.203	-2.163	0.031	*
Size × Density	decreased	-0.089	0.283	-0.315	0.752	
	increased	0.139	0.332	0.433	0.666	
Size × Microhabitat shift		-0.531	0.206	-2.574	0.011	*
Microhabitat shift × Density	decreased	0.313	0.433	0.723	0.471	
	increased	0.077	0.403	0.192	0.848	
Size × M. shift × Density	decreased	0.812	0.371	2.188	0.030	*
	increased	0.619	0.408	1.518	0.130	
