# Supplementary material for: Density‐dependent dispersal and habitat use in size‐structured populations: An experiment in wild Trinidadian guppies
Source: Ecology. 2025 Jul 18;106(7):e70151. doi: 10.1002/ecy.70151 (PMC12272143; doi:10.1002/ecy.70151)
Supplement: Supplementary file 4 — Appendix S4. [file ECY-106-e70151-s001.pdf]

Density-dependent dispersal and habitat use in size-structured populations: An experiment in wild Trinidadian guppies

Sebastiano De Bona, Karendeep Sidhu, Hanna M. Enroth & Andrés López-Sepulcre

Ecology

Appendix S3 - Alternative model for survival

The model below had an equally good fit to the model presented in the main text (ΔAIC < 4). The model includes size (standard length), sex, density expressed as a categorical variable and all two- and three-way interactions among them. Survival decreases with size in females. Survival of immature individuals is not significantly different to that of females, whereas males have significantly lower survival, which decreases with size at an even steeper slope than for females. Increased density treatment causes lower survival in females, but higher survival in males.

Table S1. GLMM for survival, including density as a categorical variable (N = 611). I = immature, M = males.

		Estimate	SE	Z value	P value	
Intercept		1.208	0.207	5.844	< 0.001	***
Size (mm)		-0.289	0.126	-2.295	0.022	*
Sex:	I	-0.867	0.481	-1.804	0.071	
	M	-2.855	0.518	-5.515	< 0.001	***
Density Treatment	decreased	0.228	0.420	0.543	0.587	
	increased	-0.739	0.253	-2.920	0.004	**
Size × sex:	I	-0.051	0.356	-0.143	0.887	
	M	-1.466	0.654	-2.243	0.025	*
Sex × Density:	I, decreased	-0.161	0.659	-0.243	0.808	
	M, decreased	-0.643	0.865	-0.743	0.457	
	I, increased	0.310	0.459	0.676	0.499	
	M, increased	1.446	0.518	2.791	0.005	**
