# Supplementary material for: Density‐dependent dispersal and habitat use in size‐structured populations: An experiment in wild Trinidadian guppies
Source: Ecology. 2025 Jul 18;106(7):e70151. doi: 10.1002/ecy.70151 (PMC12272143; doi:10.1002/ecy.70151)

Density-dependent dispersal and habitat use in size-structured populations: An experiment in wild Trinidadian guppies

Sebastiano De Bona, Karendeep Sidhu, Hanna M. Enroth & Andrés López-Sepulcre

Ecology

Appendix S4 - Discrete choice model output

Table S1. Discrete-choice model output, describing the estimated odd ratios of individuals to occupy different microhabitats. The "inflow" microhabitat is the reference against which all others are compared. Benthic area is an alternative-specific covariate, therefore its estimated effect applies to all habitats.

Coefficients	microhabitat	Estimate	SE	z-value	P-value	
(Intercept)	beach	1.167	0.899	1.298	0.194	
	core	2.318	0.530	4.372	< 0.001	***
	swamp	5.340	1.677	3.184	0.001	**
	run	6.190	1.252	4.943	< 0.001	***
Size	beach	-0.110	0.043	-2.558	0.011	*
	core	-0.090	0.024	-3.671	< 0.001	***
	swamp	-0.363	0.093	-3.888	< 0.001	***
	run	-0.373	0.068	-5.470	< 0.001	***
Sex (immature)	beach	6.400	1.194	5.359	< 0.001	***
	core	2.570	0.880	2.921	0.003	**
	swamp	4.314	1.872	2.305	0.021	*

Coefficients	microhabitat	Estimate	SE	z-value	P-value	
	run	-2.507	1.505	-1.665	0.096	
Sex (males)	beach	-0.236	3.532	-0.067	0.947	
	core	-1.172	2.461	-0.476	0.634	
	swamp	8.160	6.720	1.214	0.225	
	run	4.364	6.060	0.720	0.471	
Density	beach	0.178	3.292	0.054	0.957	
	core	0.611	2.020	0.302	0.762	
	swamp	8.027	7.367	1.090	0.276	
	run	-1.158	7.510	-0.154	0.877	
Size × sex (immature)	beach	-0.398	0.074	-5.397	< 0.001	***
	core	-0.150	0.055	-2.735	0.006	**
	swamp	-0.292	0.115	-2.545	0.011	*
	run	0.158	0.092	1.726	0.084	
Size × sex (males)	beach	0.022	0.202	0.111	0.912	
	core	0.067	0.140	0.477	0.633	
	swamp	-0.506	0.409	-1.237	0.216	
	run	-0.305	0.363	-0.840	0.401	
Size × density	beach	-0.041	0.159	-0.257	0.797	

Coefficients	microhabitat	Estimate	SE	z-value	P-value	
	core	-0.064	0.094	-0.683	0.495	
	swamp	-0.577	0.386	-1.495	0.135	
	run	0.096	0.425	0.226	0.821	
Sex (immature) × density	beach	-11.05	4.310	-2.564	0.010	*
	core	-12.692	3.200	-3.967	< 0.001	***
	swamp	-15.708	8.329	-1.886	0.059	
	run	-4.247	8.660	-0.490	0.624	
Sex (males) × density	beach	0.448	15.621	0.029	0.977	
	core	-0.248	10.743	-0.023	0.982	
	swamp	-29.467	26.34	-1.119	0.263	
	run	2.125	40.906	0.052	0.959	
Size × sex (immature) × density	beach	0.684	0.276	2.484	0.013	*
	core	0.822	0.208	3.954	< 0.001	***
	swamp	1.140	0.524	2.176	0.030	*
	run	0.331	0.540	0.612	0.540	
Size × sex (males) × density	beach	0.041	0.896	0.045	0.964	
	core	0.100	0.615	0.162	0.871	
	swamp	1.932	1.587	1.217	0.223	

Coefficients	microhabitat	Estimate	SE	z-value	P-value	
	run	-0.01	2.402	-0.004	0.997	
Benthic area		2.516	0.236	10.665	< 0.001	***

Figure S1. Predicted probability to use different microhabitats based on sex, size, and at various density treatments: control (unmodified) density, density decreased by 50%, and density increased by 50%. Immature individuals in blue, males in yellow, and females in dark green. The predictions are generated assuming all microhabitats share an equal proportion of benthic area in the pool (0.20).

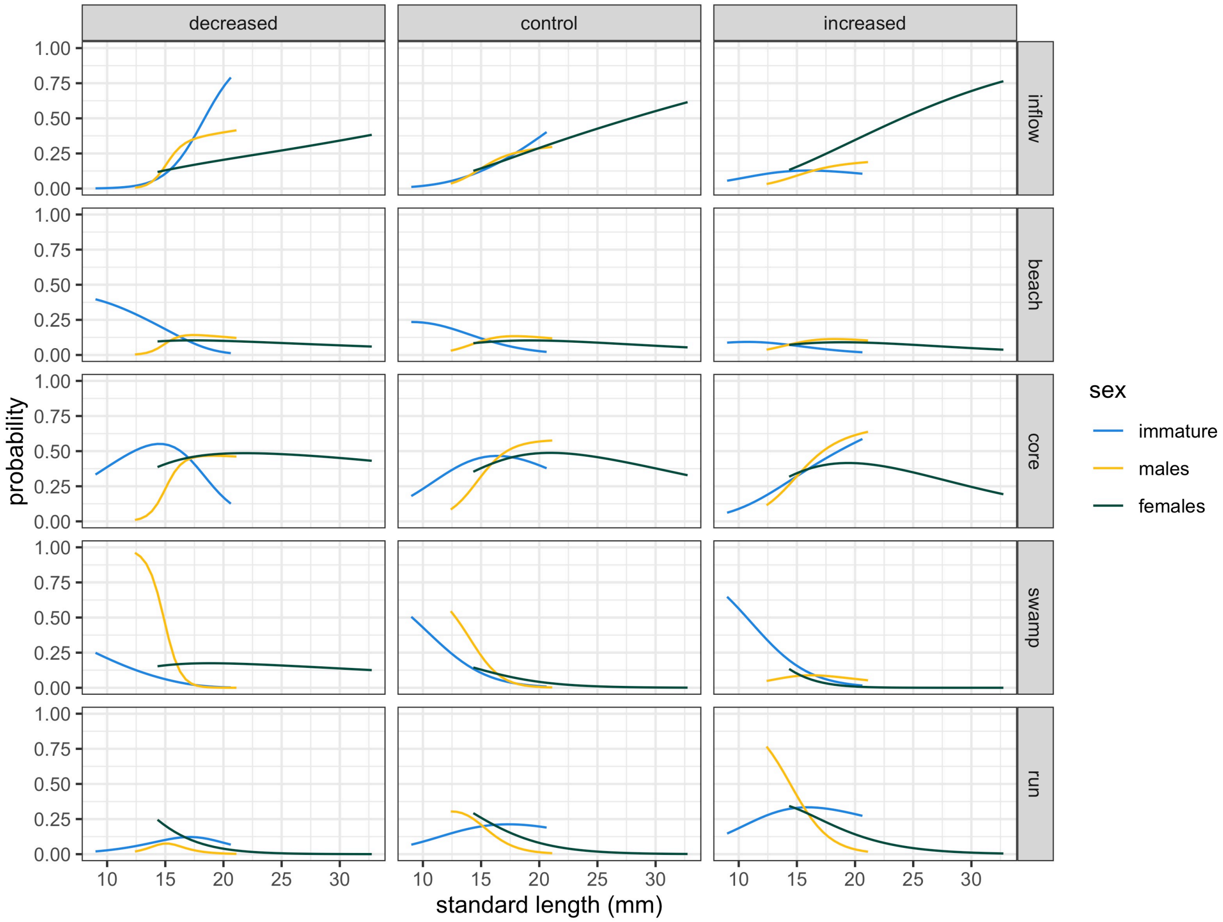

Supplement: Supplementary file 5 — Appendix S5. [file ECY-106-e70151-s004.pdf]
